# Complete Closure of a Complex Diabetic Foot Wound Following Adjunctive Treatment With a Wharton’s Jelly Mesenchymal Stromal Cell-Derived Secretome: A Case Report

**DOI:** 10.7759/cureus.117796

**Published:** 2026-10-12

**Authors:** Katherine Saborio, Jose L Fregoso Sandoval

**Affiliations:** 1 Plastic Surgery, Chloe Esthetic Center, San José, CRI; 2 Medicine, Lilova Aesthetics Institute, Ciudad Guzmán, MEX

**Keywords:** diabetes-related foot wound, diabetic foot ulcer management, mesenchymal stem cells (mscs), regenerative medicine therapies, stem cell secretome

## Abstract

Diabetes-related foot wounds remain a major clinical challenge because impaired inflammatory regulation, angiogenesis, cellular migration, and extracellular matrix remodeling can delay tissue repair and increase the risk of infection and amputation. We report the case of a 56-year-old man with type 2 diabetes mellitus who presented with a complex left foot wound of approximately two months’ duration, associated with purulent drainage, perilesional erythema, and fibrinous tissue. Wound culture yielded *Enterococcus faecalis*. Intravenous piperacillin-tazobactam, excisional debridement, wound irrigation, and serial local care achieved clinical control of the acute infectious process, although a residual open tissue defect persisted. No specific offloading strategy, specialized footwear, assistive device, or prescribed weight-bearing restriction was used during the treatment period. Adjunctive regenerative treatment with a Wharton’s jelly mesenchymal stromal cell (WJ-MSC)-derived secretome was subsequently initiated. The product was administered as a 3 mL frozen liquid formulation in 0.9% sodium chloride using a combined intralesional and perilesional technique. Three applications were performed on days 13, 39, and 69 after hospital admission. During the first treatment session, an intravenous Myers’ cocktail and high-dose vitamin C were also administered. Serial clinical photographs demonstrated progressive improvement of the wound bed, granulation tissue formation, wound contraction, and advancement of epithelialization. Complete epithelial closure was documented on day 99, corresponding to 86 days after initiation of secretome treatment and 30 days after the third application. This case documents a progressive healing response temporally associated with repeated administration of a WJ-MSC-derived secretome after clinical control of an infected diabetes-related foot wound. Although causality cannot be established from a single case, the observed clinical course is consistent with the emerging regenerative potential of MSC-derived paracrine products and supports further investigation of cell-free secretome-based strategies for difficult-to-heal diabetes-related wounds.

## Introduction

Diabetes-related foot ulcers represent a major cause of morbidity among individuals with diabetes and precede approximately 80% of diabetes-related lower-extremity amputations [1]. They are also associated with infection, hospitalization, recurrence, and increased mortality. Their management is complicated by peripheral neuropathy, vascular abnormalities, persistent inflammation, impaired angiogenesis, altered cellular function, and defective extracellular matrix remodeling [1,2]. Current international recommendations therefore emphasize comprehensive standard care, including appropriate wound debridement, infection management, offloading when indicated, assessment of perfusion, and local wound management [3].

Mesenchymal stromal cells (MSCs) have attracted increasing interest in regenerative medicine because many of their biological effects appear to be mediated through paracrine signaling. The collective products released by these cells, termed the secretome, comprise a heterogeneous mixture of soluble proteins, cytokines, chemokines, growth factors, metabolites, extracellular vesicles, and other signaling molecules capable of modulating inflammation, angiogenesis, cellular migration, proliferation, and tissue remodeling [4]. Wharton’s jelly-derived MSCs (WJ-MSCs) represent a particularly relevant cell source because of their proliferative, immunomodulatory, and paracrine properties [5].

Experimental evidence suggests that MSC-derived secretomes may enhance diabetic wound repair, although the available evidence remains predominantly preclinical and substantial heterogeneity exists among cell sources, manufacturing processes, concentrations, and administration protocols [6]. Recent experimental work specifically using secretomes derived from human WJ stromal cells has demonstrated enhanced keratinocyte and fibroblast proliferation, re-epithelialization, vascularization, and wound closure in laboratory models [7].

This report describes the clinical evolution of a complex diabetes-related foot wound treated with a WJ-MSC-derived secretome as an adjunct to conventional wound management. The case is presented to document the temporal wound-healing response and to contribute clinical evidence regarding the potential role of cell-free MSC-derived products in difficult-to-heal wounds.

## Case presentation

A 56-year-old man with a medical history of type 2 diabetes mellitus and hypertension presented with an ulcerative wound involving the left foot that had been present for approximately two months and had progressively worsened during the preceding two weeks. His documented outpatient antidiabetic treatment included metformin and glibenclamide. A blood glucose measurement of 88 mg/dL was recorded on hospital day 4. However, no HbA1c result was available in the reviewed clinical records, limiting the assessment of long-term glycemic control. Before hospital admission, the patient had received a seven-day course of intravenous levofloxacin without satisfactory clinical improvement.

On day 0, the patient was admitted for evaluation and treatment. At presentation, he was afebrile and hemodynamically stable, with a temperature of 36.0°C, blood pressure of 125/79 mmHg, heart rate of 95 beats/min, respiratory rate of 17 breaths/min, and oxygen saturation of 95% on room air.

Physical examination demonstrated deep ulcerative lesions involving the lateral aspect of the left foot, near the dorsal bases of the fourth and fifth toes, associated with purulent drainage, perilesional erythema, and fibrinous tissue. The treating diabetic foot service documented the wound classification as Wagner grade III and University of Texas grade 2, stage B (WIII/UT2B). Although the anatomical location and clinical characteristics were documented, quantitative baseline wound dimensions (length, width, and depth) and standardized serial measurements were not available in the reviewed clinical records. The clinical vascular examination documented palpable pedal and posterior tibial pulses. However, no formal peripheral sensory assessment or objective vascular measurements, such as ankle-brachial index, toe pressure, or arterial Doppler ultrasonography, were available. Therefore, peripheral neuropathy and peripheral arterial disease could not be comprehensively characterized. Plain radiography showed no radiographic evidence of osteomyelitis.

Empirical intravenous piperacillin-tazobactam at a dose of 4.5 g every six hours was initiated. A wound culture obtained during hospitalization subsequently yielded *Enterococcus faecalis*. Excisional debridement, extensive wound irrigation, and serial local wound care were performed.

During hospitalization, the infectious component progressively improved, with reduction of purulent drainage, edema, and perilesional erythema. Nevertheless, fibrinous tissue and a residual open tissue defect persisted and required continued local management. After several days of intravenous antimicrobial therapy, the patient showed substantial clinical improvement, without active purulent drainage or abscess formation, and was discharged on day 4 with instructions to continue outpatient wound care.

Adjunctive secretome treatment

Following clinical control of the acute infectious process, adjunctive regenerative treatment was initiated on day 13 after hospital admission using a WJ-MSC-derived secretome (Exogenics, Costa Rica). The decision to incorporate this treatment was based on the prolonged evolution of the wound, its previous unsatisfactory response to treatment, and the persistence of an open tissue defect despite antimicrobial therapy, excisional debridement, irrigation, and local wound care. The secretome was introduced as an adjunctive intervention rather than a replacement for conventional wound management. However, standardized measurements documenting the rate of wound healing between hospital discharge and day 13 were unavailable; therefore, inadequate healing during that specific interval could not be objectively established.

The product was supplied as a 3 mL frozen liquid formulation in 0.9% sodium chloride solution, with a reported concentration of 3X. According to the manufacturer’s certificate of analysis, the preparation was stored at −80°C until use and rapidly thawed at 37°C before administration. The secretome was delivered using a combined intralesional and perilesional technique.

The first secretome application was performed on day 13 after hospital admission. During the same treatment session, the patient also received an intravenous Myers’ cocktail and high-dose intravenous vitamin C. Myers-type intravenous micronutrient formulations described in the literature include combinations of magnesium, calcium, hydroxocobalamin, pyridoxine, dexpanthenol, B-complex vitamins, and ascorbic acid [8]. High-dose intravenous vitamin C has been evaluated at pharmacological doses of up to 110 g/m² in phase I clinical studies [9], while a broader systematic review has described intravenous doses ranging from approximately 1 g to more than 200 g per infusion across published clinical studies [10].

A second secretome application was performed on day 39, followed by a third application on day 69. The second and third treatment sessions consisted of secretome-based treatment without the same intravenous adjunctive therapies used during the first session. No local or systemic adverse events related to the secretome applications were observed or reported during the documented treatment period. Wound irrigation and serial local wound care were continued throughout the treatment period. No formal offloading intervention, specialized footwear, assistive device, or specific weight-bearing restriction was implemented during hospitalization or subsequent outpatient management.

According to the certificate of analysis, the Exogenics product consisted of a 3x concentrated secretome derived from human WJ-MSCs at passage 3. The cells were cultured in fetal bovine serum-free medium without xenogeneic additives under conditions described by the manufacturer as “GMP-like.” The conditioned medium underwent collection, filtration through a 0.22-µm membrane, and a concentration procedure described by the manufacturer as “ultracentrifugation (100 kDa).” The product corresponded to lot S260201.

Quality-control testing reported negative bacterial sterility testing after 48 hours of incubation, negative *Mycoplasma* testing by qPCR, endotoxin levels below 0.5 EU/mL by the limulus amebocyte lysate assay, and sterility testing according to USP <71>.

Photographic evolution and wound closure

Serial clinical photography documented progressive wound healing throughout the treatment course. Before initiation of secretome treatment, a residual open tissue defect persisted following antimicrobial therapy, excisional debridement, irrigation, and clinical improvement of the acute infectious process.

Following the first secretome administration on day 13, photographic assessment demonstrated progressive improvement of the wound bed, with increasing granulation tissue, reduction of the residual defect, and advancement of epithelialization. Continued wound contraction and epithelial progression were observed following the second application on day 39.

After the third secretome application on day 69, subsequent photographic follow-up demonstrated further reduction of the residual tissue defect and progressive epithelial advancement. Complete epithelial closure was documented on day 99, corresponding to 86 days after initiation of secretome treatment and 30 days after the third application.

Overall, the serial images demonstrated a progressive transition from a persistent open wound to granulation tissue formation, wound contraction, advancement of the epithelial margins, and ultimately complete epithelial closure. The serial photographic evolution of the wound is shown in Figure 1.

**Figure 1:**
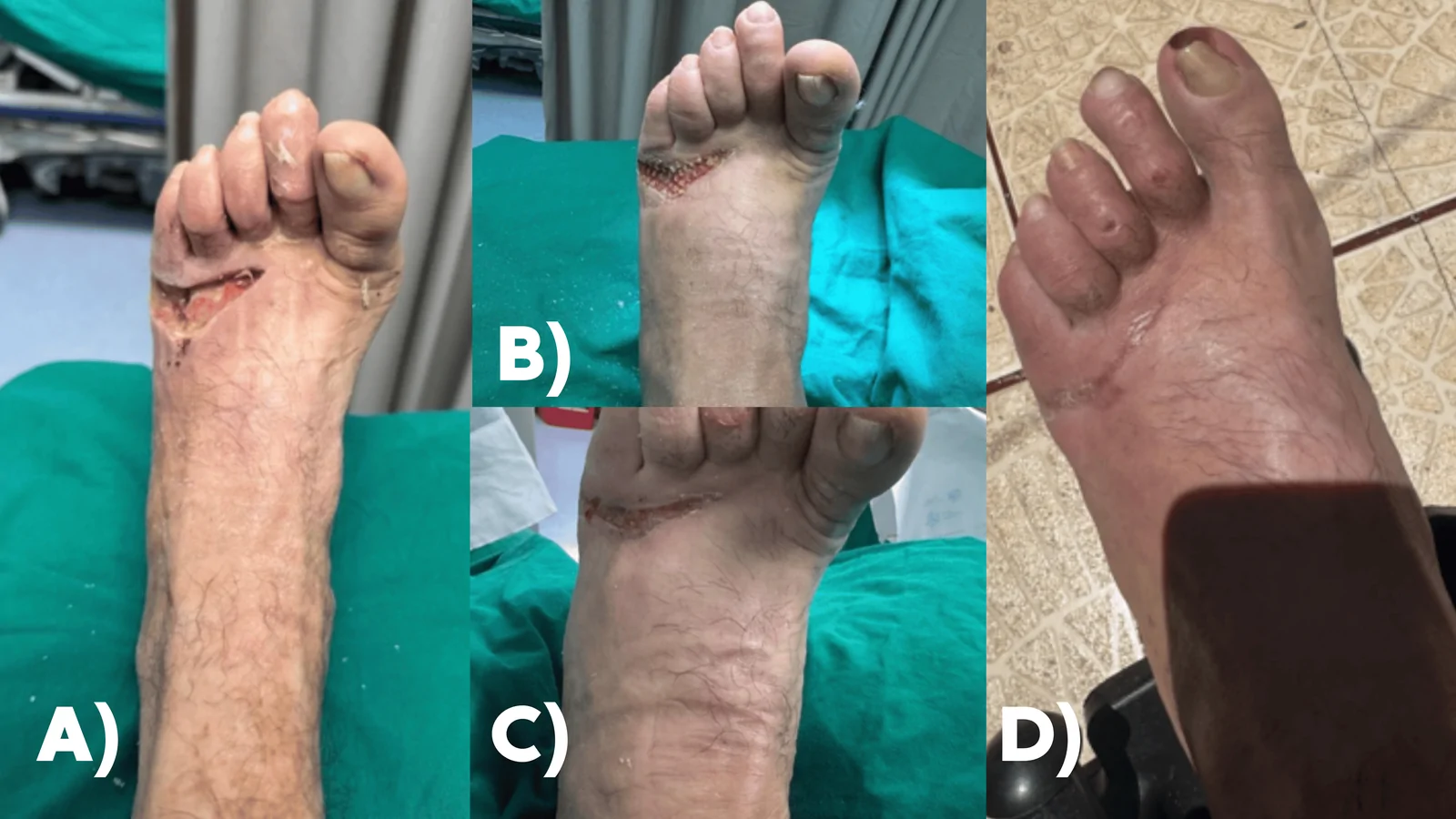
Serial clinical evolution of the diabetes-related left foot wound A) Initial residual open wound following antimicrobial therapy, debridement, irrigation, and clinical control of the acute infectious process. B) Early evolution during adjunctive secretome treatment, showing improvement of the wound bed and progressive granulation tissue formation. C) Continued reduction of the tissue defect with wound contraction and advancement of epithelialization. D) Complete epithelial closure documented on day 99, after three combined intralesional and perilesional secretome applications.

The patient’s clinical course, including hospital management, initiation of regenerative treatment, subsequent secretome applications, and complete epithelial closure, is summarized in Table 1.

**Table 1:** Clinical timeline from hospital admission to complete epithelial closure

Time point	Clinical event
Before admission	Approximately two-month history of a left foot wound with progressive worsening during the preceding two weeks; seven-day course of intravenous levofloxacin without satisfactory clinical improvement.
Day 0	Hospital admission. Wound culture obtained during hospitalization and subsequently positive for Enterococcus faecalis. Intravenous piperacillin-tazobactam, excisional debridement, wound irrigation, and serial local wound care initiated.
Day 4	Hospital discharge following substantial clinical improvement of the acute infectious process, with persistence of a residual open tissue defect.
Day 13	First secretome application using combined intralesional and perilesional administration. Concomitant intravenous Myers’ cocktail and high-dose vitamin C administered during the same session.
Day 39	Second secretome application without the same intravenous adjunctive therapies used during the first session. Continued wound irrigation and local wound care.
Day 69	Third secretome application. Serial clinical photography demonstrated continued wound contraction and epithelial advancement.
Day 99	Complete epithelial closure documented, 86 days after initiation of secretome treatment and 30 days after the third application.

## Discussion

The present case documents the progressive healing of a complex diabetes-related foot wound following the incorporation of a WJ-MSC-derived secretome into a multimodal wound-management strategy. Following antimicrobial treatment, excisional debridement, irrigation, and clinical control of the acute infectious process, a residual open tissue defect persisted. Adjunctive secretome treatment was subsequently initiated using a combined intralesional and perilesional technique, with applications performed on days 13, 39, and 69 after hospital admission. Serial clinical photographs demonstrated progressive granulation, wound contraction, and epithelial advancement, culminating in complete epithelial closure on day 99.

The relevance of this case lies in the temporal progression observed after incorporation of the regenerative intervention. Although conventional wound care had already achieved substantial control of the infectious component, tissue restoration remained incomplete. Following the introduction of secretome treatment, the wound showed a sustained pattern of granulation and re-epithelialization throughout the three applications. Importantly, the intravenous Myers’ cocktail and high-dose vitamin C were administered only during the first secretome session, whereas the second and third applications consisted of secretome-based treatment without those same intravenous adjuncts. Continued wound progression after the subsequent applications strengthens the clinical interest of the temporal association, although it does not establish a direct causal relationship.

This clinical course is biologically consistent with the growing understanding that many regenerative effects of MSCs are mediated by paracrine signaling rather than direct cellular engraftment. The MSC secretome contains a complex mixture of cytokines, chemokines, growth factors, angiogenic mediators, soluble proteins, metabolites, regulatory nucleic acids, and extracellular vesicles that may simultaneously influence several phases of wound repair [4,5]. These biological signals may modulate inflammatory activity, promote fibroblast and keratinocyte proliferation and migration, support angiogenesis, and contribute to extracellular matrix remodeling and epithelial restoration [4,5].

These mechanisms are especially relevant in diabetes-related wounds, where healing is impaired by persistent inflammation, dysfunctional angiogenesis, altered fibroblast activity, delayed keratinocyte migration, and abnormal extracellular matrix remodeling [1,2]. In this context, a cell-free product derived from WJ-MSCs may offer a biologically attractive regenerative strategy because it acts on several disrupted pathways through paracrine signaling rather than relying on a single molecular mechanism.

Preclinical evidence supports this concept. Suhandi et al. reported in a systematic review and meta-analysis of 20 in vivo studies involving 382 animals with diabetic wounds that stem cell-derived secretomes were associated with a significant improvement in wound closure and reductions in neutrophil and macrophage infiltration [6]. These findings suggest that secretome-based therapies may influence both the inflammatory and proliferative phases of diabetic wound repair [6].

Evidence specifically involving WJ-derived secretome is particularly relevant to the present case. Chin et al. demonstrated that secretome obtained from human WJ-derived stromal cells enhanced cellular proliferation and wound closure in vitro and promoted re-epithelialization, vascularization, granulation tissue maturation, and wound repair in more complex experimental models [7]. The biological effects described in these models parallel several of the clinical changes documented photographically in the present patient, particularly progressive granulation, contraction of the tissue defect, and epithelial advancement.

Emerging human evidence further supports the regenerative potential of WJ-MSC-derived paracrine products. Kishta et al. reported a randomized controlled clinical trial in which WJ-MSC-derived exosomes were used as adjunctive treatment for persistent diabetic foot ulcers, with improved healing outcomes compared with standard treatment [8]. Although exosomes and whole secretome are biologically distinct products, this clinical evidence supports the broader hypothesis that cell-free paracrine products derived from WJ-MSCs may improve diabetic wound repair.

The product used in the present case was a 3X concentrated secretome derived from human WJ-MSCs at passage 3. According to the certificate of analysis, the cells were cultured in fetal bovine serum-free medium without xenogeneic additives, and the final preparation underwent filtration and microbiological quality-control testing. Sterility testing was negative, Mycoplasma testing by qPCR was negative, and endotoxin levels were below 0.5 EU/mL. These characteristics are relevant because manufacturing conditions and product quality may substantially influence secretome composition and biological activity [9].

Another feature of this case is the combined intralesional and perilesional administration technique. The secretome was administered as a 3-mL frozen liquid formulation in 0.9% sodium chloride, maintained at −80°C until use and rapidly thawed at 37°C before combined intralesional and perilesional administration. From a biological perspective, this approach may facilitate exposure of the wound bed and adjacent viable tissue to paracrine mediators. However, optimal dosing, concentration, injection pattern, treatment interval, and route of administration of WJ-MSC-derived secretomes in diabetic wounds have not yet been standardized. Therefore, the three-session regimen used in this patient should be interpreted as a documented clinical protocol rather than an established therapeutic standard.

The progressive healing trajectory observed throughout repeated secretome administration is clinically noteworthy. Although the sequential changes suggest sustained tissue repair, the relative contribution of the secretome cannot be distinguished from that of ongoing conventional wound care. Nevertheless, the observed progression supports further investigation of repeated secretome administration as an adjunctive regenerative strategy.

The case also illustrates a broader trend within regenerative medicine toward cell-free therapeutic approaches. Compared with direct cellular administration, secretome-based products could theoretically reduce concerns related to cell survival, engraftment, uncontrolled differentiation, and some aspects of the complexity of cell handling, while retaining biologically active paracrine mediators [4,5]. Nevertheless, the clinical translation of these products depends on improved standardization of manufacturing, composition, potency, dosing, and quality-control parameters.

The main limitations of this report are its single-patient design, the absence of a comparator group, the contribution of conventional wound care, and the concomitant intravenous therapies administered during the first treatment session. In addition, standardized prospective planimetry and comprehensive molecular characterization of the secretome were not available. These factors prevent attribution of the clinical outcome exclusively to the secretome. However, they do not negate the value of the observed temporal sequence, especially because progressive healing continued during the second and third secretome applications without the same intravenous adjunctive therapies [10].

Overall, this case provides human clinical documentation of progressive wound repair associated with repeated administration of a WJ-MSC-derived secretome after control of an acute diabetic foot infection. The clinical evolution is consistent with experimental evidence demonstrating pro-regenerative, pro-angiogenic, immunomodulatory, and epithelial-reparative effects of MSC-derived secretomes [11-12]. Although the findings remain hypothesis-generating, they support further clinical investigation of WJ-MSC-derived secretomes as adjunctive cell-free regenerative therapies for difficult-to-heal diabetes-related wounds [13].

## Conclusions

This case documents the complete epithelial closure of a complex diabetes-related foot wound following the incorporation of a WJ-MSC-derived secretome into a multimodal wound-management strategy. After clinical control of the acute infectious process with antimicrobial therapy, excisional debridement, irrigation, and serial wound care, the patient received three combined intralesional and perilesional secretome applications on days 13, 39, and 69 after hospital admission. Serial clinical photography demonstrated progressive granulation, contraction of the wound defect, and epithelial advancement, culminating in complete epithelial closure on day 99, 30 days after the third application.

The temporal relationship between repeated secretome administration and progressive wound healing is clinically noteworthy and is consistent with the regenerative, immunomodulatory, pro-angiogenic, and epithelial-reparative effects described for MSC-derived paracrine products. Although a single case cannot establish therapeutic efficacy or causality, the observed clinical course supports further investigation of WJ-MSC-derived secretomes as adjunctive cell-free regenerative therapies for difficult-to-heal diabetes-related wounds. Prospective studies with standardized product characterization, dosing and administration protocols, objective wound measurements, comparator groups, and longer follow-up are required to determine reproducibility and clinical benefit.
